# Creation of an engineered APC system to explore and optimize the presentation of immunodominant peptides of major allergens

**DOI:** 10.1038/srep31580

**Published:** 2016-08-19

**Authors:** Sandra Rosskopf, Sabrina Jutz, Alina Neunkirchner, Martín R. Candia, Beatrice Jahn-Schmid, Barbara Bohle, Winfried F. Pickl, Peter Steinberger

**Affiliations:** 1 Institute of Immunology, Center for Pathophysiology, Infectiology and Immunology, Medical University of Vienna, Vienna, Austria; 2 Department of Pathophysiology and Allergy Research, Center for Pathophysiology, Infectiology and Immunology, Medical University of Vienna, Vienna, Austria

## Abstract

We have generated engineered APC to present immunodominant peptides derived from the major aero-allergens of birch and mugwort pollen, Bet v 1_142–153_ and Art v 1_25–36_, respectively. Jurkat-based T cell reporter lines expressing the cognate allergen-specific T cell receptors were used to read out the presentation of allergenic peptides on the engineered APC. Different modalities of peptide loading and presentation on MHC class II molecules were compared. Upon exogenous loading with allergenic peptides, the engineered APC elicited a dose-dependent response in the reporter T cells and the presence of chemical loading enhancers strongly increased reporter activation. Invariant chain-based MHC class II targeting strategies of endogenously expressed peptides resulted in stronger activation of the reporters than exogenous loading. Moreover, we used Bet v 1 as model allergen to study the ability of K562 cells to present antigenic peptides derived from whole proteins either taken up or endogenously expressed as LAMP-1 fusion protein. In both cases the ability of these cells to process and present peptides derived from whole proteins critically depended on the expression of HLA-DM. We have identified strategies to achieve efficient presentation of allergenic peptides on engineered APC and demonstrate their use to stimulate T cells from allergic individuals.

Accessory signals provided by antigen presenting cells (APC) govern the responses of T cells towards cognate peptide-major histocompatibility complex (MHC) molecules. Attempts to manipulate T cells *in vivo* as well as the *in vitro* generation of T cells to be used for adoptive transfer critically depends on our knowledge of signals that enhance or efficiently inhibit T cell responses. In this context much can be learned from studies on the interaction of natural APCs such as dendritic cells (DC) with T cells but these cells also harbor certain constraints. Due to the plethora of activating and inhibitory ligands provided by professional APC it is difficult to study the role of individual costimulatory or coinhibitory ligands using such cells. In addition, the limited availability of MHC-matched donors and variability in their T cell stimulatory capacity are of concern when using primary APC to study T cell activation processes.

The use of engineered antigen presenting cells (eAPC) - often also designated artificial APCs - is an attractive option to stimulate antigen-specific T cells since it allows to provide T cells with accessory signals of choice. The human erythroleukemia cell line K562 is an ideal platform for antigen presentation to human T cells as it can be furnished with MHC molecules of choice but is devoid of endogenously expressed MHC class I as well as class II (MHCII) molecules, thereby minimizing the stimulation of allo-reactive T cells^1^. Initial studies have focused on the generation and use of MHC class I expressing K562 cells to stimulate CD8^+^ T cells specific for antigens derived from pathogens or tumors^2^,^3^,^4^,^5^. More recently these cells have been shown to be suitable to present MHCII restricted antigens to CD4^+^ T cells. In this context the focus was also on the stimulation of CD4^+^ T cells recognizing peptides derived from viruses or tumor antigens^6^,^7^. To date such cells have not been used to study CD4^+^ T cells that contribute to pathological processes. In this context eAPC might be useful to identify signals that efficiently dampen helper T cells that drive aberrant immune responses.

Allergen-specific Type 2 helper (Th2) CD4^+^ T cells play a central role in initiating and promoting type I allergy^8^. By inducing class switching of B cells via IL-4 they are responsible for the production of allergen-specific IgE, the major effector molecule in this disease. In addition, they produce IL-13 and IL-5 thereby stimulating airway epithelial cells and eosinophils^9^,^10^. Th2 cells also contribute to late phase reactions^8^. Consequently, allergen-specific Th2 CD4^+^ T cells are primary targets in attempts to ameliorate IgE-associated allergic disease^11^ and improved knowledge regarding signals that dampen Th2 responses is desirable.

Studies on allergen-specific T cell clones have yielded invaluable information on immunodominant T cell epitopes of major allergens present in pollen extracts or other allergen sources^12^,^13^. Importantly, such clones have been used to isolate cDNAs encoding allergen-specific T cell receptors (TCRs) making it possible to reconstruct the “allergen-specific synapse” at the molecular level^14^,^15^,^16^. This is a valuable tool for pursuing and testing strategies to counteract Th2 based allergen-specific T cell responses^15^. They have been used to demonstrate that regulatory T cells and Th1 cells recognizing peptides derived from allergens might reduce symptoms in allergic individuals by directly antagonizing Th2 cells or via other mechanisms^15^,^17^. eAPC stably expressing MHCII molecules of choice are valuable for studying mechanisms and strategies for antigen processing and presentation to CD4^+^ T cells. Moreover, they might be useful tools to expand and study allergen-specific T cells derived from allergic individuals. Accessory molecules like coinhibitory ligands of choice can be expressed on these cells. Consequently they can be used to identify signals that inhibit allergen-specific T cells or skew them towards a non-Th2 phenotype.

Here we report on the use of K562 cells stably expressing MHCII molecules to present immunodominant T cell epitopes from allergens. Jurkat-based T cell reporter cells transgenic for allergen-specific TCRs were applied as functional read-out to analyze and compare different strategies to achieve efficient presentation of allergenic peptides on human MHCII molecules. We have analyzed different modalities to exogenously load K562-eAPC with allergenic peptides and whole allergens. In addition, we have explored approaches to target endogenously expressed immunodominant peptides and full-length allergen proteins into the MHCII presentation pathway.

## Results

### Generation of allergen-specific engineered antigen presenting cells and allergen-specific T cell reporter cells

**Figure 1:**
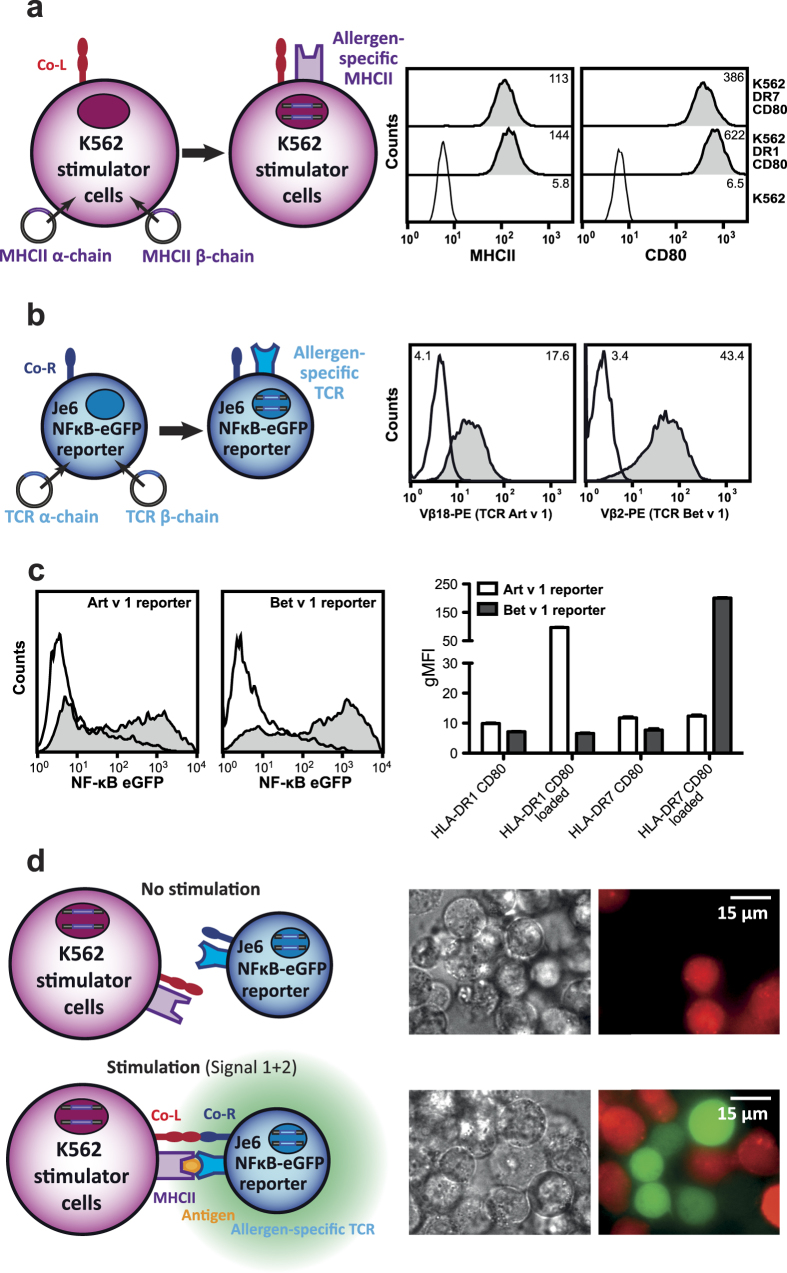
Generation and characterization of allergen-specific T cell stimulator cells and allergen-specific T cell reporter cells. (**a**) Scheme illustrating the generation of K562 cell-based engineered APC (left) and expression analysis of surface molecules on these cells by flow cytometry (right). Open histograms: K562 cells; filled histograms: respective engineered APC. (**b**) Scheme illustrating the generation of Jurkat-based allergen-specific T cell reporter cells (left) and expression analysis of the allergen-specific TCRs using antibodies specific for the Vß-chains of the respective transgenic TCR (right). Open histograms: Staining of allergen-specific reporter cells with isotype control antibody; filled histograms: staining with antibodies specific for the transgenic TCR-Vß chain. (**c**) Activation of Je6 NF-κB reporters with allergen-specific stimulator cells harboring the appropriate restriction element in absence (open histograms) or presence (grey histograms) of allergenic peptides Art v 1_23–36_ or Bet v 1_142–153_ (left). T cell reporter specific for Art v 1 and Bet v 1 were cocultured with eAPC expressing the indicated molecules in presence or absence of allergenic peptides. Mean gMFI ± SD of triplicates is shown and experiment is representative for three independently performed experiments. (**d**) Reporter gene-expression and formation of immunological synapses visualized by cell imaging using chamber slides. K562 stimulator cells expressing HLA-DRB1 (transduced with mCherry for microscopic visualization) are cocultivated with Je6 NF-κB-eGFP-reporter cell line without and with allergenic peptide addition (upper and lower panel, respectively).

We aimed to establish eAPC as stimulator cells that can be used to present allergenic peptides to allergen-specific T cells. The major birch pollen allergen Bet v 1 and the major mugwort pollen allergen Art v 1, which are both well-characterized on the molecular level, were used as model allergens in our studies^12^,^13^,^18^. The MHCII molecules HLA-DRB1^*^01:01 and HLA-DRB1*07:01 were expressed on the human K562 erythroleukemia cell line along with CD80, a potent costimulatory ligand (Fig. 1a).

A Jurkat JE6.1 NFκB-eGFP T cell line was engineered to express a previously described T cell receptor (TCR)^14^, which recognizes the single immunodominant T cell epitope Art v 1_25–36_ presented on HLA-DRB1^*^01:01 (Art v 1 reporter cells). We also generated Bet v 1 reporter cells expressing a TCR, that interacts with the immunodominant peptide Bet v 1_142–153_, in the context of HLA-DRB1*07:01^15^. Antibodies specific for the β-chain of the respective TCR variable regions were used to select TCR-transgenic Jurkat reporter cell clones (Fig. 1b). These eAPCs and TCR-transgenic Jurkat-reporter T cells were cocultured for 24 hours in presence of immunodominant peptides and reporter induction (eGFP-expression) was measured by flow cytometry. Reporter gene expression was only induced in presence of the cognate allergenic peptide and eAPC expressing the appropriate restriction element (Fig. 1c). The formation of immunological synapses and reporter-gene expression was visualized by life cell imaging (Fig. 1d). These experiments demonstrated functionality and specificity of our eAPC - NFκB-eGFP T cell-reporter system.

### Effects of peptide concentration and costimulation

**Figure 2:**
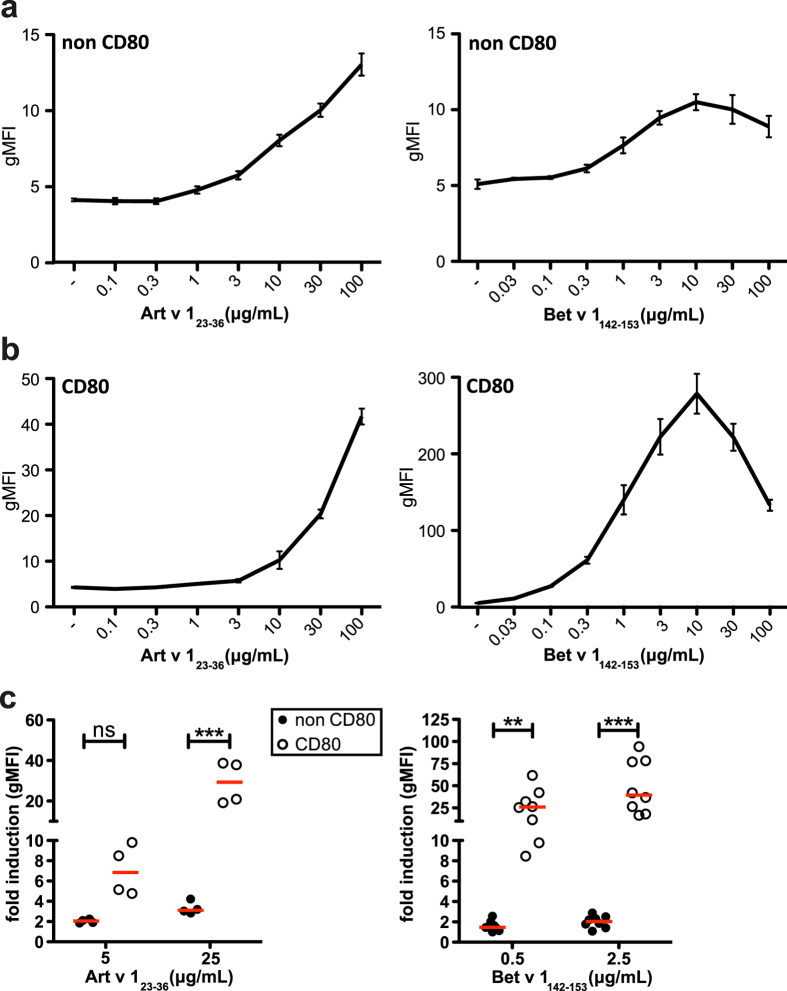
Effects of peptide concentration and CD80 costimulation. (**a **+ **b**) Art v 1 specific (left) and Bet v 1 specific (right) T cell reporter cells were cocultured with CD80^−^ eAPC (**a**) or CD80^+^ eAPC (**b**) expressing the appropriate restriction elements in the presence of allergenic peptides used at the indicated concentrations. Mean gMFI ± SD of triplicates is shown. (**c**) Effect of CD80 costimulation on Art v 1 and Bet v 1 reporter activation using Art v 1_23–36_ or Bet v 1_142–153_ peptide at the indicated concentrations. Mean fold induction is shown for duplicate values and median fold induction of all experiments is indicated as line. Statistics by two-way ANOVA, followed by Bonferroni post-test (*P ≤ 0.05; **P ≤ 0.01; ***P ≤ 0.001; ns not significant).

Next, we tested the reporter T cells by adding different amounts of peptide ranging from 0.03 μg/mL to 100 μg/mL to the cocultures (exogenous loading). A dose-dependent response was measured in both reporters. While the Art v 1 reporter did not reach a plateau of stimulation, exogenous loading with 10 μg/mL of allergenic peptide resulted in a maximum response in the Bet v 1 reporter (Fig. 2a). The higher sensitivity of the Bet v 1 reporter cells might be owed to the better expression of the allergen-specific TCR on these cells (Fig. 1b). For further experiments we used 5 and 0.5 μg/mL peptide as a standard concentration for the Art v 1 and Bet v 1 reporters, respectively.

To study the effects of costimulation in our eAPC system, we established single cell clones expressing the costimulatory ligand CD80 and corresponding CD80^−^ cell clones, which had comparable MHCII expression (Suppl. Fig. 1a,1b). Coculture with CD80^+^eAPCs also resulted in a dose-dependent response (Fig. 2b). For direct comparison, two peptide concentrations were evaluated for each model allergen, namely 5 and 25 μg/mL for Art v 1_23–36_ and 0.5 and 2.5 μg/mL for Bet v 1_142–153_. In the presence of CD80 costimulation, a significant enhancement of the reporter activation (shown as fold induction) was evident (Fig. 2c). We also assessed whether coinhibitory signals would reduce the response to antigen. PD-1 was expressed on the Jurkat T cell reporters and its ligands PD-L1 and PD-L2 on the respective eAPC (Suppl. Fig. 1c). Significantly reduced activation of the reporters upon PD-1/PD-L1 or PD-L2 engagement was measured. Addition of a blocking antibody specific for PD-1 significantly reverted this effect (Suppl. Fig. 1d). Taken together, our data indicate that eAPC induce a dose-dependent response to allergenic peptides, which can be modulated upon expression of costimulatory or coinhibitory ligands.

### Endogenous loading strategies

**Figure 3:**
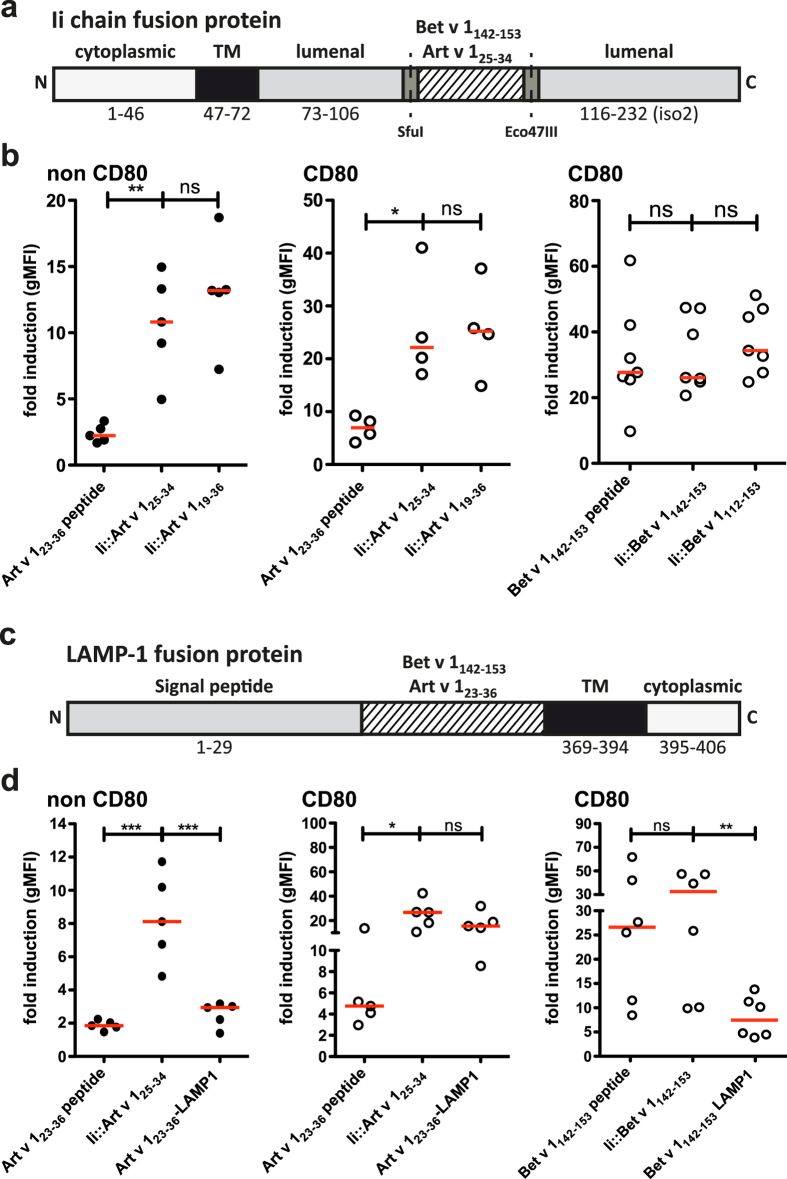
Comparison of endogenous loading strategies: epitope variation, Ii chain and LAMP-1. (**a**) Schematic illustration of fusion proteins of the invariant chain and allergenic peptides. (**b**) Comparison of exogenous and endogenous MHCII loading strategies via addition of allergenic peptides or expression of invariant chain (Ii) fusion proteins in the eAPC, respectively. 5 μg/mL Art v 1_23–36_ and 0.5 μg/mL Bet v 1_142–153_ peptide were used were used for exogenous loading. Mean fold induction is shown for duplicate values and median fold induction of all experiments is indicated as line. Statistics by one-way ANOVA, followed by Tukey’s multiple comparison post-test (*P ≤ 0.05; **P ≤ 0.01; ***P ≤ 0.001; ns not significant). (**c**) Schematic illustration of fusion proteins of LAMP-1 (lysosomal-associated membrane protein 1) and allergenic peptides. (**d**) Comparison of exogenous and endogenous MHCII loading strategies via peptide addition and Ii or LAMP1 fusion proteins. 5 μg/mL Art v 1_23–36_ and 0.5 Bet v 1_142–153_ peptide were used for exogenous loading. Mean fold induction is shown for duplicate values and median fold induction of all experiments is indicated as line. Statistics by one-way ANOVA, followed by Tukey’s multiple comparison post-test (*P ≤ 0.05; **P ≤ 0.01; ***P ≤ 0.001; ns not significant); (*TM: transmembrane domain*).

In addition to the exogenous loading of immunodominant peptides, we assessed strategies in which allergen-derived peptides were endogenously expressed in eAPC. The respective peptides were targeted towards MHCII presentation. First, stimulator cells expressing fusion proteins of the invariant chain (CD74, Ii) and the immunodominant peptides were created. In these constructs the genetic information for the class II-associated invariant chain peptide (CLIP) was exchanged for the sequence of the immunodominant T cell epitope to constantly supply MHCII molecules with antigenic peptide (Fig. 3a)^14^,^19^. In addition to Ii-fusion proteins harboring the immunodominant T cell epitopes Art v 1_25–34_ and Bet v 1_142–153_, we also tested variants containing longer sequences derived from Art v 1 and Bet v 1. We generated an Ii-fusion protein harboring an extended Art v 1_19–36_ peptide for which increased binding to HLA-DRB1^*^01:01 was reported^20^. We also created an Ii-fusion protein containing a longer sequence (Bet v 1_112–153_) that includes another frequently recognized T cell epitope (Bet v 1_112–123_)^12^. Single cell clones of the respective eAPCs were established and equivalent MHCII density, Ii and CD80 expression was confirmed by flow cytometry (Suppl. Fig. 2a).

In case of Art v 1 we observed significantly higher activation of the reporter cells upon stimulation with eAPC expressing Ii-fusion proteins compared to eAPC exogenously loaded with peptides. This effect was observed in the presence or absence of CD80-costimulation. There was a tendency that eAPCs endogenously loaded with the longer Art v 1 peptide (li::Art v 1_19–34_) had a higher capacity to induce reporter activation than eAPC expressing li::Art v 1_25–34_, but the difference was not statistically significant (Fig. 3b). In the case of Bet v 1, eAPC exogenously loaded with Bet v 1_142–153_ and eAPC expressing Ii-fusions of allergenic peptide both induced very strong reporter activation and there was no significant difference between exogenous and endogenous (invariant chain based) loading strategies. Stimulator cells expressing the longer Bet v 1-derived peptide (112–153) were as efficient in activating the reporter T cells specific for Bet v 1_142–153_ as eAPC expressing the shorter version Ii::Bet v 1_142–153_ (Fig. 3b).

Another approach for targeting antigenic peptides to the MHCII presentation pathways had been demonstrated by Wu *et al.* by fusing the HPV-16 E7 protein to LAMP1 (lysosomal-associated membrane protein 1)-sequences^21^,^22^,^23^. That approach targeted the protein into the lysosomal compartments. To compare Ii-based loading with LAMP1-based strategies we generated and expressed constructs encoding Art v 1_23–36_ and Bet v 1_142–153_ fused to the signal peptide, the transmembrane domain and the cytosolic domain of LAMP-1 (Fig. 3c). Both reporter T cell lines were specifically stimulated by eAPC expressing these LAMP1-fusion proteins but stimulator cells expressing the respective Ii-fusion proteins induced stronger reporter gene activation (Fig. 3d). This indicated that the Ii-based targeting is a superior strategy to induce MHCII presentation of antigenic peptides.

### Endogenously expressed peptides are refractory to displacement by competitor peptides

**Figure 4:**
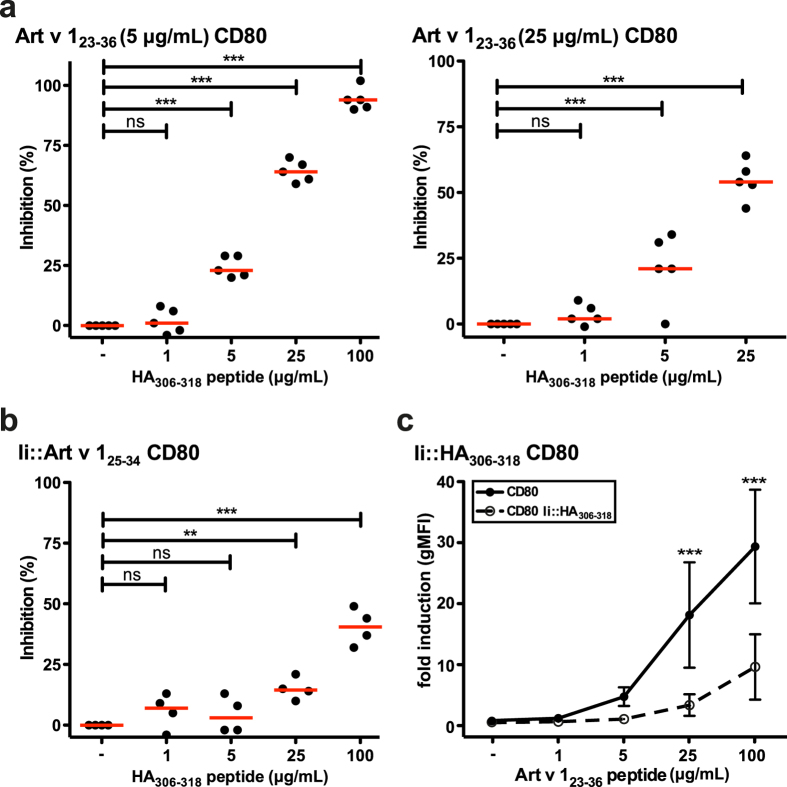
Competition for MHCII loading. (**a**) Art v 1 reporter T cells were stimulated with Art v 1_23–36_ peptide alone or in presence of indicated amounts of competitor peptide (HA_306–318_ peptide). Inhibition of stimulation is shown for duplicate values and median inhibition of five experiments is indicated as line. Statistics by one-way ANOVA, followed by Tukey’s multiple comparison post-test (*P ≤ 0.05; **P ≤ 0.01; ***P ≤ 0.001; ns not significant). (**b**) Effect of HA_306–318_ peptide addition as competitor for the presentation of endogenously loaded allergenic Art v 1 peptide. Inhibition of stimulation is shown for duplicate values and median inhibition of five experiments is indicated as line. Statistics by one-way ANOVA, followed by Tukey’s multiple comparison post-test (*P ≤ 0.05; **P ≤ 0.01; ***P ≤ 0.001; ns not significant). (**c**) Art v 1 reporter cells were stimulated with Art v 1 peptide in the presence of eAPC endogenously expressing competitor peptide (Ii::HA_306–318_) or control eAPC. Mean fold induction of duplicates ± SD of five experiments is shown. Statistics by two-way ANOVA, followed by Bonferroni post-test (*P ≤ 0.05; **P ≤ 0.01; ***P ≤ 0.001; ns not significant).

To assess the effect of competing peptides the HA_306–318_ peptide with high affinity for HLA-DRB1*01:01 was used as a competitor for the presentation of the allergenic Art v 1_23–36_ peptide. Upon exogenous loading the response of the reporter cells to Art v 1_23–36_ was inhibited by the competitor peptide in a concentration-dependent fashion (Fig. 4a). At high concentration (100 μg/mL), the HA_306–318_ peptide almost completely inhibited the response to 5 μg/mL Art v 1 peptide (Fig. 4a). By contrast the response to endogenously expressed Art v 1 peptide was only diminished by 40% in the presence of 100 μg/mL competitor peptide (Fig. 4b). Moreover, we observed that exogenous loading of eAPC expressing HA_306–318_ peptide-Ii fusion protein with Art v 1_23–36_ peptide resulted in greatly reduced stimulation of the reporter cells compared to “empty” DR1 CD80 stimulator cells (Fig. 4c). As above, eAPC were matched for equivalent MHCII density, Ii and CD80 expression (Suppl. Fig. 2b). These results corroborate that Ii-targeting results in efficient loading of peptides and that the latter are not prone to displacement by exogenous peptides.

### Factors influencing peptide loading onto MHCII

**Figure 5:**
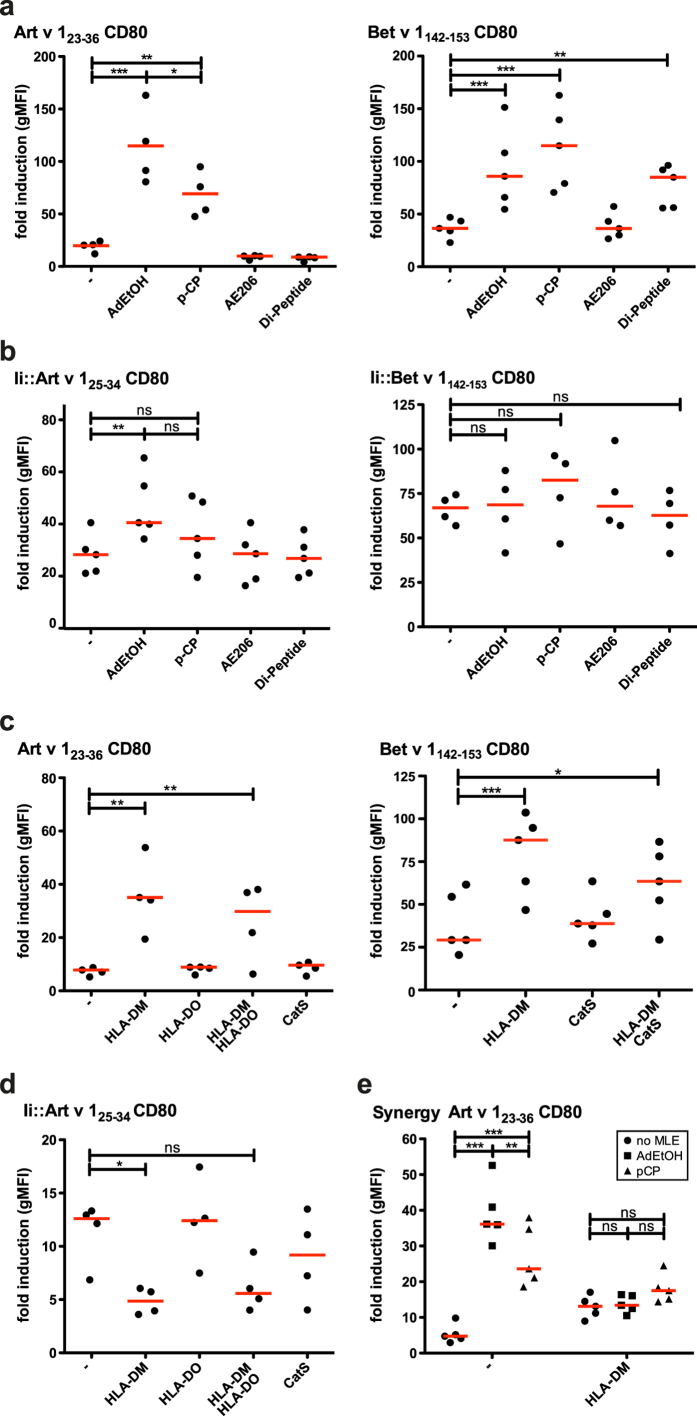
Factors influencing peptide loading - MHC loading enhancers and peptide processing machinery. (**a **+ **b**) Effect of MHCII loading enhancers (MLE) on exogenous (**a**) or endogenous (**b**) peptide loading. Art v 1 (left) or Bet v 1 (right) specific T cell reporters were stimulated with eAPC exogenously loaded with peptide or endogenously loaded with immunodominant peptides expressed as invariant chain fusion proteins without MLE or in presence of MLE. (**c**) Influence of molecules involved in MHCII antigen processing on exogenous peptide loading. Art v 1 (left) or Bet v 1 (right) specific T cell reporters were stimulated with allergenic peptides in presence of eAPC overexpressing the indicated molecules. (**d**) Influence of molecules involved in MHCII antigen processing on endogenous loading. Art v 1 specific T cell reporters were stimulated with eAPC expressing Art v 1 peptides as invariant chain fusion protein alone or in combination with the indicated molecules. (**e**) Evaluation of the synergy between HLA-DM and non-peptide MLEs. Art v 1 specific reporter cells were stimulated with Art v 1 peptide in the presence of control eAPC or eAPC overexpressing HLA-DM. MLE were added as indicated. Statistics by two-way ANOVA, followed by Bonferroni post-test (*P ≤ 0.05; **P ≤ 0.01; ***P ≤ 0.001; ns not significant). Art v 1_23–36_ and Bet v 1_142–153_ peptides were used at 5 and 0.5 μg/ml for exogenous loading, respectively. The non-peptide MLEs 2-(1-adamantyl) ethanol (short AdEtOH) and p-chlorophenol (short p-CP) were used at concentrations of 100 μM and 1000 μM, respectively. The peptide-MLEs AE206 (NH_2_-LRLKLPK-COOH) and Di-Peptide (Ac-NH-FR-CONH_2_) were evaluated at a concentration of 150 μM. Mean fold induction is shown for duplicate values and median fold induction of multiple experiments is indicated as line. Statistics (**a**–**d**) by one-way ANOVA, followed by Tukey’s multiple comparison post-test (*P ≤ 0.05; **P ≤ 0.01; ***P ≤ 0.001; ns not significant).

Binding of peptides to MHCII can be increased by small molecules called MHC loading enhancers (MLE)^24^,^25^, which could thus be of use to amplify immune responses. The non-peptide MLE 2-(1-adamantyl)ethanol (AdEtOH) and p-chlorophenol (p-CP), as well as the peptide- MLE AE206 (NH_2_-LRLKLPK-COOH) and Di-Peptide (Acetyl-NH-FR-CONH_2_) were assessed regarding their ability to influence exogenous and endogenous peptide loading. The non-peptide MLE (AdEtOH and p-CP) dramatically increased the response of both reporter cells indicating that these compounds are highly efficient in improving MHCII loading of exogenously added allergenic peptides. DR1 and DR7 both harbor a glycine on position 86 of the HLA-DRβ chain and thus meet the requirements necessary for AdEtOH mediated loading enhancement^24^. Interestingly, AdEtOH was the more efficient MLE for DR1-mediated presentation of Art v 1 peptide whereas Bet v 1 peptide presentation on DR7 was more strongly enhanced by p-CP. For the peptide-MLE a significant effect was only observed with the Di-Peptide in case of Bet v 1_142–153_ (Fig. 5a). Of note, the functional effects of MLE were reflected in binding assays (data not shown). Loading of endogenously expressed peptides was also enhanced by both non-peptide MLE but the effect was less pronounced. Both peptide-MLE did however not affect presentation of endogenously expressed peptides (Fig. 5b). For both model allergens similar results were obtained when testing the effects of MLE using CD80^−^eAPC (data not shown). In additional experiments we tested the effect of non-peptide MLE for conditions, in which peptides were preloaded for 3 hours on the eAPC followed by a washing step to avoid the presence of unbound peptides in the stimulation cultures. Peptide preloading resulted in reduced presentation of allergenic peptides compared to standard peptide loading conditions. The addition of MLE during peptide preloading greatly increased the capacity of eAPC to present allergenic peptides to the reporter cells (Suppl. Fig. 2c).

Processing of antigens for MHCII presentation is a complex process, involving multiple proteins. We investigated the influence of molecules critically involved in MHCII antigen processing on exogenous and endogenous peptide loading. HLA-DM, HLA-DO, HLA-DM together with HLA-DO and Cathepsin S (CatS) were overexpressed in the Art v 1 stimulator cells. qPCR was used to measure expression of the respective mRNAs in our eAPC. K562 cells did not express significant amounts of these molecules endogenously, however, cells transduced with the respective constructs contained high mRNA levels (Suppl. Table 1)^26^. Overexpression of HLA-DM led to a significantly increased response to exogenous peptides in both model systems. HLA-DM was detected on the surface of our eAPC (data not shown) and previous work has demonstrated a functional role of surface HLA-DM in antigen presentation^27^,^28^,^29^. We found that enhanced presentation of exogenous peptide upon expression of HLA-DM was greatly reduced by addition of an HLA-DM antibody. Moreover, we observed that blocking of acidification in endosomal/lysosomal compartments using chloroquine did not abrogate HLA-DM effects (Suppl. Fig. 3c). These results suggest that surface HLA-DM mediates enhanced peptide loading in our system. We did not observe any significant effects upon overexpressing HLA-DO or CatS (Fig. 5c). Notably, MHCII presentation of allergenic peptides expressed as Ii-fusion proteins was greatly reduced upon expression of HLA-DM. In contrast, HLA-DO or CatS did not have a significant effect on reporter activation in response to endogenously expressed allergenic peptides (Fig. 5d). Since HLA-DM expression as well as the presence of non-peptide MLE were effective in increasing MHCII loading of exogenous peptides we evaluated the synergistic potential of HLA-DM with non-peptide MLEs for Art v 1_23–36_. While addition of 100 μM AdEtOH or 1000 μM p-CP significantly increased peptide presentation in the absence of HLA-DM, these MLE did not significantly enhance the stimulatory activity of eAPC overexpressing HLA-DM (Fig. 5e).

### Proliferation of allergen-specific T cells in response to engineered APCs

**Figure 6:**
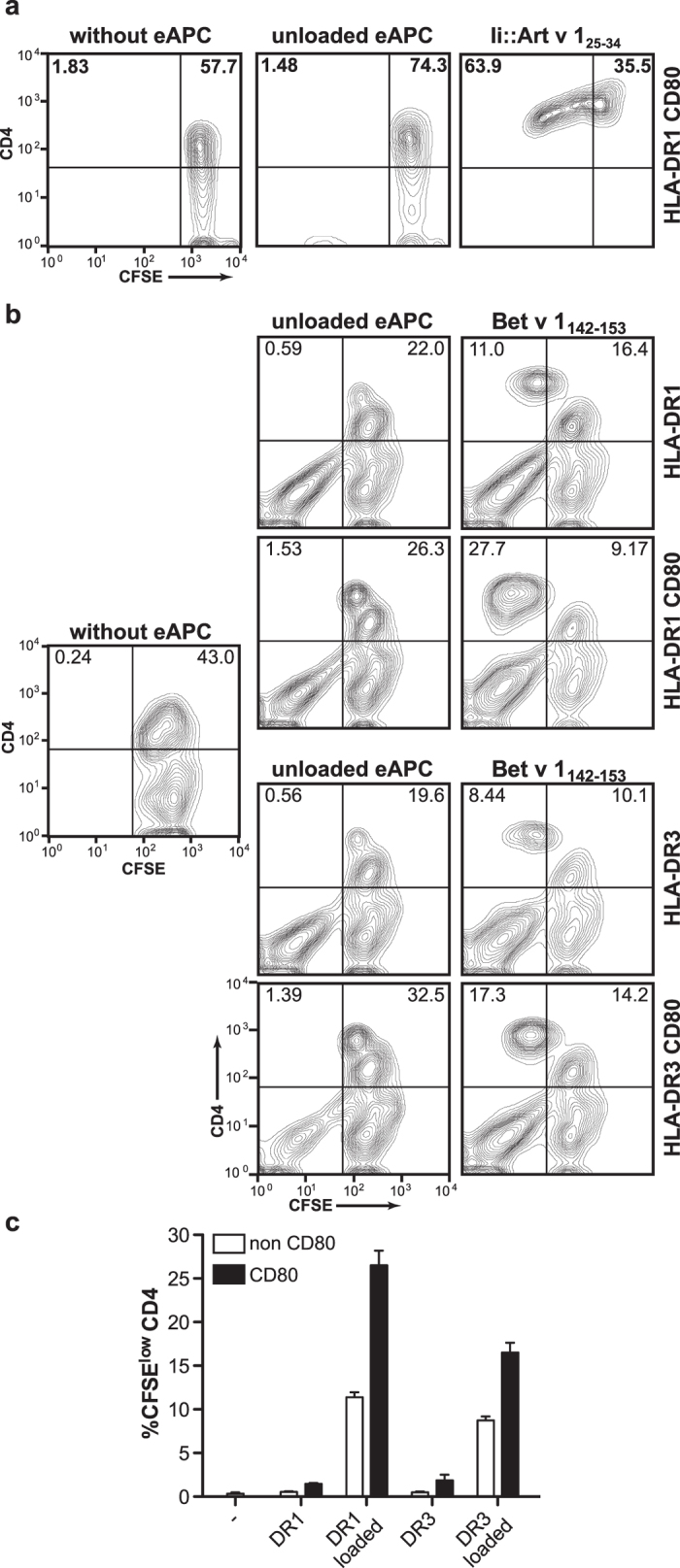
Proliferation of allergen-specific T cells in response to engineered APCs. (**a**) A CFSE-labeled Art v 1-specific T cell clone was cocultured with eAPC peptide expressing HLA-DRB1 and CD80. eAPC endogenously loaded via invariant chain fusion proteins with Art v 1_25–34_ or unloaded eAPC were used and T cells cultured without stimulus were used as additional control. After 4 days of coculture cells were harvested, stained for CD4 expression and analyzed by flow cytometry. Data is representative for six tested Art v 1-specific TCC. (**b**) A CFSE-labeled Bet v 1-specific T cell line (derived from a HLA-DRB1:01^+^ and HLA-DRB1:03^+^ donor) was cocultured with eAPC peptide expressing restriction elements and CD80 as indicated. eAPC preloaded with Bet v 1_142–153_ or unloaded APC were used and T cells cultured without stimulus were used as additional control. Following 5 days of coculture cells were harvested, stained for CD4 expression and analyzed by flow cytometry. Cell population in the CFSE^low^CD4^−^ gate represent eAPCs. (**c**) Mean and SD of CFSE^low^CD4^+^ T cells from duplicate wells of (**b**) are shown. The experiment was repeated with similar outcome.

To demonstrate that eAPC can also be used to efficiently stimulate allergen-specific T cells we performed CFSE-dilution experiments with T cell clones (TCC) and T cell lines (TCL) established from HLA-DR-matched allergic donors. Irradiated eAPC expressing Ii-targeted immunodominant Art v 1_25–34_ peptide induced strong proliferation in Art v 1-specific TCCs (Fig. 6a and data not shown). This response was strictly dependent on the presence of antigen since this cell line did not respond to eAPC not expressing the immunodominant peptide (Fig. 6a). In additional experiments, we tested a Bet v 1-specific TCL from PBMCs of a donor (HLA-DRB1*01 and HLA-DRB1*03) allergic to birch pollen allergen. The immunodominant Bet v 1 epitope (142–153) is bound by several HLA-DR molecules including HLA-DRB1*01 and HLA-DRB1*03^30^. Consequently we tested this TCL with two different eAPC expressing the respective MHCII molecules preloaded with 0.5 μg/mL Bet v 1_142–153_ peptide in the presence of 100 μM AdEtOH as MLE. Irradiated stimulator cells were then cocultivated for 5 days with CFSE-labeled donor T cells. These experiments again demonstrated that eAPC can be used to efficiently stimulate allergen-specific CD4^+^ T cells since a high fraction of CFSE^low^ CD4^+^ T cells were detected in cocultures of allergen-loaded eAPC expressing DR1 or DR3 but not in cocultures with unloaded eAPC (Fig. 6b,c). Presence of the costimulatory ligand CD80 on the eAPC increased the percentage of CD4^+^ T cells that had proliferated in response of immunodominant peptide (Fig. 6b,c).

### Devising eAPCs efficiently presenting whole allergens

**Figure 7:**
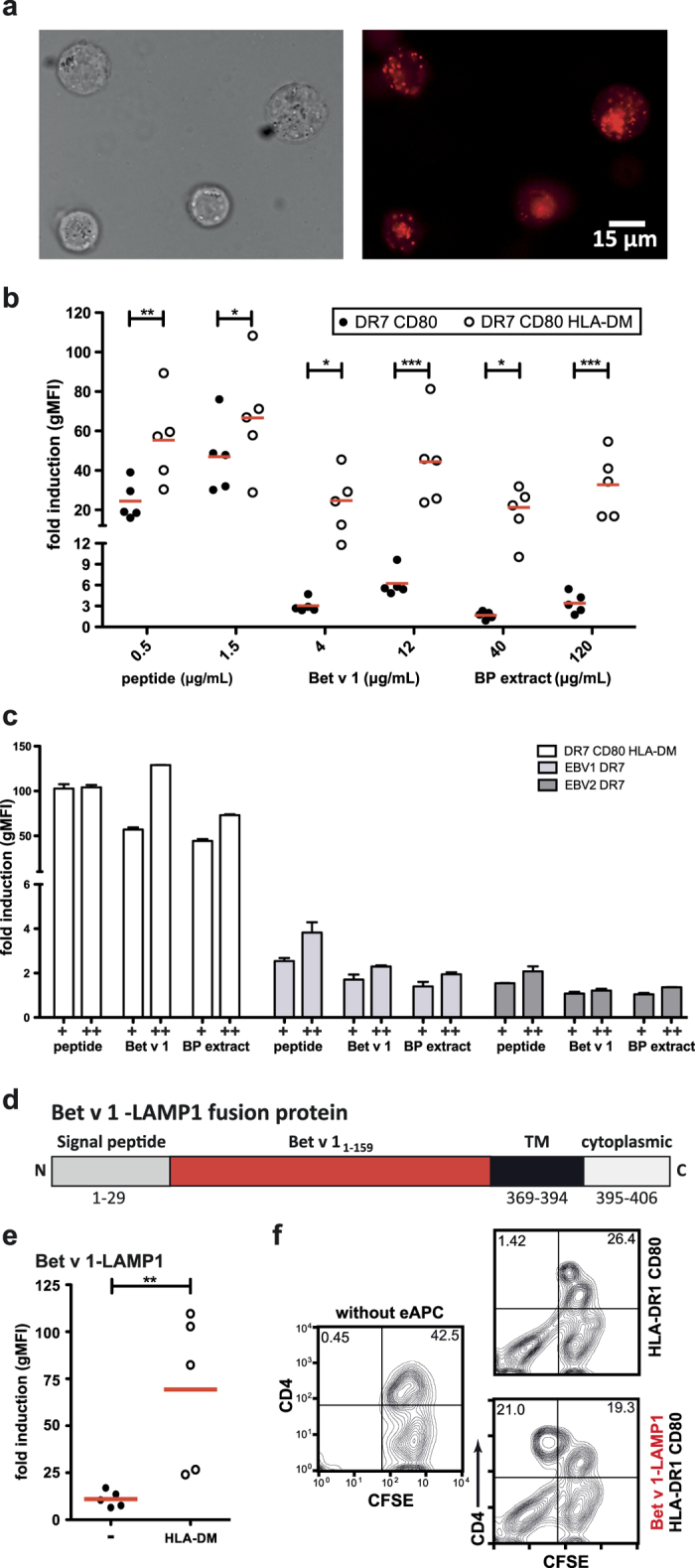
Devising eAPCs efficiently presenting whole allergens. (**a**) Uptake transport to lysosomal compartments of Bet v 1 coupled to pHrodo^®^ visualized by fluorescence microscopy. Sections of microscopic pictures representative for multiple experiments are shown. (**b**) Effect of HLA-DM on the capacity of eAPCs to present allergenic peptides and to process whole allergen for MHCII presentation. Bet v 1 specific T cell reporters were cocultured in presence of control eAPC and eAPC expressing HLA-DM. Allergenic peptide Bet v 1_142–153_, Bet v 1 or birch pollen (BP) extract was added at the indicated concentrations. Mean fold induction is shown for duplicate values and median fold induction of multiple experiments is indicated as line. Statistics by two-way ANOVA, followed by Bonferroni post-test (*P ≤ 0.05; **P ≤ 0.01; ***P ≤ 0.001; ns not significant). (**c**) Comparison of the stimulatory capacity of eAPCs and EBV cell lines using Bet v 1 specific T cell reporters (antigen source as indicated in (**b**)). Mean fold induction ± SD is shown for duplicate values. (**d**) Schematic illustration of a fusion protein of LAMP1 (lysosomal-associated membrane protein 1) and the whole Bet v 1 allergen; (TM … transmembrane domain). (**e**) Effect of HLA-DM on the reporter activation using stimulator cells harboring the Bet v 1-LAMP1 fusion protein. eAPC expressing the Bet v 1-LAMP1 fusion protein and eAPC coexpressing the Bet v 1-LAMP1 fusion protein and HLA-DM were used to stimulate Bet v 1 specific T cell reporter cells. Mean fold induction is shown for duplicate values and median fold induction of multiple experiments is indicated as line. Statistics by Mann-Whitney test (*P ≤ 0.05; **P ≤ 0.01; ***P ≤ 0.001; ns not significant). (**f**) A CFSE-labeled Bet v 1-specific T cell line was cocultured with eAPC expressing HLA-DRB1*01 and CD80. eAPC expressing the Bet v 1-LAMP1 fusion protein or unloaded eAPC were used. Following 5 days of coculture, cells were harvested, stained for CD4 expression and analyzed by flow cytometry.

Major allergens like Bet v 1, harbor multiple relevant T cell epitopes recognized by allergic individuals^12^. Consequently, the availability of eAPC that efficiently process whole allergens would be advantageous. We used Bet v 1 as model allergen to evaluate the potential of eAPCs to take-up and process full-length proteins for the presentation of immunodominant peptides. The uptake into the lysosomal compartments was analyzed using rBet v 1 coupled to pHrodo^TM^, a dye, which shows bright fluorescence when entering into acidic compartments. Bet v 1 was readily taken up and transported to lysosomal compartments (Fig. 7a). DR7^+^CD80^+^eAPCs were loaded with Bet v 1_142–153_ peptide, full-length Bet v 1 or birch pollen extract and tested in reporter assays. We observed that eAPC were inefficient in presenting allergenic peptides derived from the full-length Bet v 1 protein, thus indicating a defect in processing or MHCII loading of antigens: Compared to Bet v 1_142–153_ peptide-loaded cells the eAPC loaded with Bet v 1 or birch pollen extract had a dramatically reduced capacity to stimulate allergen-specific reporter cells. Since K562 cells do not express significant amounts of HLA-DM, we analyzed whether expressing HLA-DM in our eAPC would improve their capability to present antigenic peptides from whole allergens. We observed that HLA-DM^+^eAPC showed a dramatic improvement in their capability to present allergenic peptides derived from full-length Bet v 1 or birch pollen extracts (Fig. 7b).

In a next step, we compared the stimulatory capacity of eAPCs (HLA-DRB1*07, CD80 and HLA-DM) with that of HLA-DRB1*07^+^EBV cell lines. Independent of the antigen source eAPC had a superior capability to stimulate Bet v 1-specific T cell reporters (Fig. 7c). APCs were analyzed for MHCII density, CD80 and CD58 expression and a higher expression of CD80 was observed in eAPC (Suppl. Fig. 3a).

In order to evaluate eAPC, which express whole allergens endogenously, we generated a fusion construct of LAMP1 and the whole Bet v 1 molecule (Fig. 7d). eAPC expressing this construct induced moderate activation of Bet v 1-specific reporter T cells. Coexpression of HLA-DM dramatically enhanced the stimulatory capacity of eAPC expressing the LAMP1-Bet v 1 fusion protein (Fig. 7e). Single cell clones of the respective eAPCs were checked for equivalent MHCII density, CD80 and HLA-DM expression. Expression of the LAMP1-fusion construct was confirmed with a mAb specific for Bet v 1_73–103_ (Suppl. Fig. 3b)^31^. CFSE-dilution experiments demonstrated that Bet v 1-LAMP1^+^eAPC were highly efficient in specifically stimulating a T cell line established from a DR1^+^-birch pollen allergic patient (Fig. 7f).

## Discussion

In this study we developed a stable system of engineered APC for efficient presentation of allergenic peptides to allergen-specific human CD4^+^ T cells. We have used two distinct model allergens, Art v 1 and Bet v 1, the major allergens of mugwort and birch pollen, respectively. Art v 1 is a unique allergen as it contains only one major epitope (Art v 1_25–36_), which is predominantly recognized in the context of HLA-DRB1*01^13^. Thus, it is a highly suitable candidate allergen to be presented on eAPC since around 70% of the mugwort allergic individuals can be covered with a single peptide-MHC combination. By contrast, Bet v 1 the second allergen used in this study contains several relevant peptides in addition to the immunodominant T cell epitope Bet v 1_142–156_, which was shown to be recognized by more than 60% of the T cell lines generated from birch pollen allergic individuals^12^. Moreover, allergenic Bet v 1 peptides including the immunodominant epitope are presented to allergen-specific T cells in the context of different MHCII molecules^18^,^20^,^32^.

The use of eAPC offers a wide variety of possibilities to target antigens for MHC presentation. Consequently, a sensitive and robust readout for the presence of immunodominant peptide-MHC complexes on the surface of our APC was required to identify efficient strategies and protocols for immunodominant T cell epitope loading and presentation on MHCII molecules. To this end we have generated stable T cell reporter lines expressing previously described human TCR recognizing Art v 1_25–36_ and Bet v 1_142–156_ in the context of HLA-DRB1*01 and HLA-DRB1*07, respectively^14^,^15^. These reporters are a reliable and robust read-out for presentation of allergen-specific peptides and they can also be used to gauge the impact of accessory signals like stimulatory or coinhibitory signals on T cell responses.

In addition to exogenous loading of our eAPC, we endogenously expressed allergenic peptide as Ii-fusion proteins to target them for MHCII expression. This approach is a very efficient way to deliver antigen-specific signals to specific T cells and offers several advantages: it can be used to efficiently express longer antigenic sequences and can thus be used to introduce more T cell epitopes in the eAPC. Moreover, it allows for a continuous, high-level expression of allergen-specific peptide-MHCII complexes, and we show that endogenously produced peptides are largely refractory to displacement by exogenous competitor peptides. This is a considerable advantage in approaches that aim at efficient presentation of a particular antigen.

MHCII loading is influenced by many exogenous and cell intrinsic elements and in this study we have identified factors that efficiently enhance allergen presentation on eAPC. Regarding the effect of MLE we found that the non-peptide MLE AdEtOH and p-CP both strongly increased the response of reporters to eAPC pulsed with allergenic peptides, whereas only minor effects have been observed with the peptide-MLE that were analyzed. Professional APC express various molecules that are involved in antigen processing and MHCII loading. We observed that overexpression of HLA-DM in K562-based eAPC had a strong impact on their ability to present allergenic peptides to T cells. It strongly enhanced the response of Art v 1 and Bet v 1 reporters upon exogenous peptide pulsing. HLA-DM has a complex and multilayered role in the loading of MHCII molecules^33^. Stabilizing “empty” class II dimers in their open, peptide receptive state is one of its functions and this ability might critically contribute to the enhanced loading of exogenous allergenic peptides onto MHCII molecules that we have observed in this study^34^. Chemical MLE also promote the peptide-receptive form of MHCII molecules and MLE like p-CP are often referred to as “chemical analogues” of HLA-DM^24^,^25^. In light of their redundant functions regarding the presentation of exogenous peptide on MHCII it is thus not surprising that there was not a significant additive effect between HLA-DM and MLE.

The importance of HLA-DM was even more pronounced when analyzing their ability to present allergenic peptides from whole allergens. Bet v 1 is readily taken up by K562 cells and is targeted to endolysosomal compartments but efficient presentation of peptides from whole Bet v 1 or birch pollen extract was only observed in APC engineered to express HLA-DM. In addition to allergenic peptides expressed as Ii-fusion proteins, we expressed the whole Bet v 1 molecule as a LAMP-1 fusion protein. This approach also leads to efficient presentation of allergenic Bet v 1 peptides by eAPC. Notably we found contrasting effects of HLA-DM in different strategies to endogenously express allergenic peptides in K562-derived eAPC: whereas presence of HLA-DM strongly improved the presentation of Bet v 1 peptides from LAMP1-Bet v 1 fusion proteins it dramatically reduced the presentation of Ii-fused allergenic peptides. HLA-DM catalyzes the displacement of the Ii peptide (CLIP) from nascent MHCII molecules and it is thus not surprising that HLA-DM also acts to displace Ii-targeted antigenic Art v 1_25–36_ since this peptide does not have a high affinity for HLA-DR1^20^,^33^. Collectively, our results indicate that expression of HLA-DM enhances the efficiency of K562 based eAPC to present peptides derived from allergenic proteins except for Ii-fused sequences. Moreover, these data imply that LAMP-1 based strategies are the method of choice to target whole allergens for MHCII presentation. Evidently, engineering APC to present epitopes from whole allergens is advantageous since most allergens contain several T cell epitopes. However, it is well established that MHC class II presented peptides are also generated outside of endosomes and it is thus possible that some epitopes might not survive processing in endosomes and would be lost upon targeting allergens into these compartments^35^,^36^.

HLA-DM greatly accelerates the peptide exchange on MHCII molecules thereby inducing a selection process favoring the presentation of high-affinity peptides^37^,^38^. Thus, expression of HLA-DM in eAPC is an important measure to facilitate the presentation of immunodominant peptides from whole allergens or allergen-extracts to allergen-specific CD4^+^ T cells. Although we found that HLA-DM was sufficient for the efficient presentation of the major allergenic peptide from whole Bet v 1 it is conceivable that additional molecules will be required for efficient presentation of other allergens. Many different proteases contribute to the generation of a large variety of peptides that can be loaded onto MHCII^35^,^39^. Nevertheless, using a cell free system Hartman *et al.* demonstrated that a very limited set of molecules is sufficient to achieve presentation of immunodominant epitopes^40^,^41^. Based on their data, it can be expected that expression of Cathepsin S, L and B will endow HLA-DM^+^eAPC with the ability to present immunodominant epitopes from a broad variety of antigens and allergens.

Antigen-loaded EBV-immortalized B cells are widely used as an inexhaustible source of autologous APC for the generation and stimulation of antigen- or allergen-specific T cell lines and clones. We found that compared to EBV lines, eAPC that coexpress HLA-DM and CD80 are clearly superior in presenting allergenic peptides to Bet v 1-specific reporter T cells. We intent to use our eAPC to stimulate primary allergen-specific CD4^+^ T cells derived from individuals with IgE-associated allergies. We will study the impact of distinct accessory signals on these cells and our long-term goal is to identify pathways that can be exploited to inhibit allergen-specific T cells or to counteract the pathological Th2 phenotype. Here, we demonstrated that eAPC expressing different restriction elements can be used to efficiently stimulate allergen-specific T cell lines generated from individuals allergic to Art v 1 or Bet v 1. To our knowledge this is the first report on the use of stable eAPC to activate human allergen-specific CD4^+^ T cells derived from allergic donors. There is a wide range of additional applications for eAPC - allergen-specific reporter systems. They are reliable tools to study mechanisms of allergen processing and presentation and could be used to develop *in vitro* bioassays to measure the T cell activity to various allergen sources including allergen-containing food.

## Methods

### Cell culture, antibodies and flow cytometry

The human Jurkat T cell line JE6.1 and the human K562 cell line and derivatives thereof were cultured as described elsewhere^42^. EBV cell lines were generated from HLA-typed donors as described previously^43^. Recombinant Bet v 1.0101 allergen was purchased from Biomay AG (Vienna, Austria). Art v 1_23–36_, Bet v 1_142–153_ and HA_306–318_ peptides were purchased from Thermo Fisher (Waltham, MA, USA) at a purity of >80%. Allergenic extract preparation was performed as described elsewhere^44^. The following monoclonal antibodies were used to confirm surface expression on or intracellular expression in engineered K562 cells and Jurkat reporter cell lines: CD80-PE (2D10), PD-L1-PE (29E.2A3), PD-L2-PE (24F.10C12), PD-1-PE (EH12.2H7), HLA-DM-PE (MaP.DM1), CD4-PE (OKT4), MHCI-APC (W6/32) CD28-APC (CD28.2) and appropriate isotype control all purchased from Biolegend (San Diego, CA). HLA-DR-PE antibody (L243) was purchased from BD Bioscience (San Jose, CA). TCRβ chain-specific mAbs anti-TCR Vβ2-PE (MPB2D5, for Bet v 1_142–153_ specific TCR) and anti-TCR Vβ18-PE (BA62.6, for Art v 1 specific TCR) were purchased from Beckmann-Coulter (Marseille, France). Intracellular expression of the invariant chain (Ii) or fusion proteins of the Ii with immunodominant T cell epitopes in the K562 cells was detected with an in-house produced mouse-anti-human CD74 antibody (5–329) in conjunction with DyLight^TM^ 649-conjugated goat anti-mouse IgG (H + L) antibodies (Jackson ImmunoResearch Laboratories, Inc., West Grove, PA). A mAb specific for Bet v 1_73–103_ was used for detection of the Bet v 1-LAMP1 fusion protein^31^. A purified functional grade PD-1 (EH12.2H7; LEAF^TM^) antibody was used for blocking studies (Biolegend). Staining of intracellular proteins was performed using the BD Cytofix/Cytoperm^TM^ kit according to the manufacturers´ recommendations.

Flow cytometry analysis was performed using a FACSCalibur™ flow cytometer (BD Bioscience). FlowJo software (version 10.0.6., Tree Star, Ashland, OR) was used for data analysis.

### Expression constructs and retroviral transduction

Expression cassettes encoding previously described fusion proteins of Bet v 1, Art v 1 and influenza with invariant chain sequences (Ii fusion proteins) were cloned into the retroviral vector pCJK2^14^,^19^,^45^. Retroviral expression constructs encoding PD-L1, PD-L2, CD80 have been previously described^45^. Retroviral expression constructs encoding HLA-DMα; HLA-DMβ, HLA-DOα; HLA-DOβ, HLA-DRA, HLA-DRB1*01:01, HLA-DRB1*07:01 and Cathepsin S were generated by excising the cDNAs encoding these molecules from pEAK12 expression plasmids and cloning them into the retroviral vector pCJK2. Expression constructs encoding fusion proteins of human lysosomal associated membrane protein 1 (LAMP-1)^46^ with the whole Bet v 1 molecule or with allergenic peptides derived from Bet v 1 or Art v 1 have been generated by PCR overlap extension using expression plasmid encoding Bet v 1 (kindly provided by B. Kratzer) and a cDNA library derived from human MNC as templates^47^. The LAMP1 constructs were cloned into a retroviral expression vector pSandy (a derivative of the pCJK2 vector produced in-house) using SfiI restriction sites. The integrity of expression constructs generated for this study was confirmed by DNA-sequencing. The ecotropic receptor was expressed in K562 cells and Jurkat-NF-κB-eGFP reporter cells to use retroviral particles pseudotyped with the ecotropic envelope to stably express molecules of interest in these cell lines using a previously described protocol^48^. Amino acid sequences of the molecules used for designing the LAMP-1 expression constructs were derived from the following molecules: Art v 1 (UniProtKB - Q84ZX5), Bet v 1 (UniProtKB - O23748), LAMP-1 (UniProtKB - P11438).

### Generation of engineered APC

eAPC expressing HLA-DRB1*01:01, HLA-DRB1*03:01 and HLA-DRB1*07:01 were generated by coexpressing HLA-DRα and the respective HLA-DR-β-chain. In addition, eAPC coexpressing HLA-DRB1*01:01, HLA-DRB1*03:01 and HLA-DRB1*07:01 with CD80 were generated. Selected eAPC were further engineered to endogenously express MHC class II targeted allergenic peptides or whole allergens by retroviral transduction with constructs encoding fusion protein of allergens with the Ii or with LAMP-1. eAPC coexpressing invariant chain fusion proteins and PD-ligands (PD-L1 or PD-L2) were also generated. Single cell clones were established after each transduction step to assure homogenous and comparable expression of the respective molecules.

### Generation of allergen-specific T cell reporters

Allergen-specific T cell reporters are based on a previously described Jurkat (Je6) NF-κB-eGFP-reporter cell line generated in our laboratory^49^. The α-chain and β-chain of the TCRs specific for the allergenic epitopes Art v 1_25–36_ and Bet v 1_142–153_ were retrovirally (pMMp412 vector) coexpressed in the reporter cells^14^,^15^. Single cell clones were established by limiting dilution and checked for transgenic TCR expression using antibodies specific for the respective Vβ-chains. Single cell clones were selected based on the expression of allergen-specific TCR and their capability to specifically respond to allergenic peptides. For evaluation of coinhibitory pathways, human PD-1 was retrovirally expressed in the T cell reporters.

### Cocultivation experiments of stimulator cells with reporter T cell lines

eAPCs (3 × 10^5^/well) were treated with allergenic peptides (5 μg/mL Art v 1_23–36_ or 0.5 μg/mL Bet v 1_142–153_) or mock-treated and cocultured with the allergen-specific Jurkat reporter T cell lines (5 × 10^5^/well) in 96-well flat bottom plates in a final volume of 100 μl, unless indicated otherwise. Addition of allergenic peptides to the co-cultures is referred to as “exogenous loading” throughout the manuscript. In additional experiments eAPC were pre-loaded for three hours at 37 °C in standard cell culture medium followed by a washing step prior to initiation of cocultures with T cell reporter cells. Stimulation with 1 μg/mL PMA and 1 μg/mL Ionomycin (both from Sigma Aldrich, St. Louis, MO) was used as positive control. After 24 h, cells were harvested and stained with an MHC class I-APC antibody. Reporter gene expression was measured using a FACSCalibur™ flow cytometer (BD Biosciences). Geometric mean of fluorescence intensity (gMFI) was determined for each sample to calculate median and standard deviation of the duplicate or triplicate wells. eAPC (MHC class I negative) were excluded from the analysis. For most experiments, reporter gene induction in response to allergenic peptide was normalized to eGFP expression in non-stimulated reporters and expressed as fold-induction of the geometric mean fluorescence intensity (gMFI).

For competition assays, immunodominant peptide and competitor peptide were added simultaneously at the indicated concentrations.

### Visualization of immunological synapses with life cell imaging

eAPCs (4 × 10^5^/well), retrovirally transduced to express mCherry, were cocultivated with Je6 NF-κB-eGFP-reporter cell lines (2.4 × 10^5^/well) expressing the Art v 1_25–36_-specific TCR in the 8 well-Nunc™ Lab-Tek™ Chamber Slide System (Thermo Fisher) in RPMI cell culture medium for 24 h. For allergen-specific stimulation 5 μg/mL Art v 1_23–36_ peptide were used for exogenous HLA-DR1 loading. Analysis was performed on a Leica DMI4000 B light microscope using LAS AF software (Leica Microsystems, Wetzlar, Germany) and ImageJ (NIH, Bethesda, MD).

Recombinant Bet v 1 coupled to pHrodo^®^ (kindly provided by C. Kitzmüller) was used to assess endocytosis in K562 cells. eAPCs (2 × 10^5^/well) were incubated with 5 μg/mL rBet v 1-pHrodo in RPMI cell culture medium for 24 h. Microscopic analysis was performed as above mentioned.

### MHC class II loading enhancers

In indicated experiments MHCII loading enhancers (MLE) were added to the cocultures or during pre-loading of eAPC. As non-peptide MLEs 2-(1-adamantyl)ethanol AdEtOH (HLA-DRB1 specific, Sigma-Aldrich)^24^ and p-chlorophenol p-CP (pan-MLE, Thermo Fisher)^25^ were used at 100 μM, 1000 μM, respectively. Further, the two peptide-MLEs AE206 (NH_2_-LRLKLPK-COOH)^50^ and Di-Peptide (Ac-NH-FR-CONH_2_)^51^ were evaluated at 150 μM each. The peptide-MLEs were produced by Thermo Fisher at a purity of >80%.

### T cell line proliferation assay with eAPCs

Approval from the ethics committee of the Medical University of Vienna was obtained for studies with primary human cells (EK1538/2014) and informed consent was obtained from the volunteer donors. All methods were carried out in accordance with the approved guidelines. Allergen-specific T cell lines (TCL) were generated from PBMCs of allergic donors as described^52^. An Art v 1-specific TCL generated from PBMCs of a mugwort pollen allergic donor (HLA-DRB1:01^+^) was labeled with CFSE as described^53^. Irradiated eAPC expressing the respective MHCII molecules and eAPCs coexpressing MHCII molecules and fusion proteins of Art v 1_25–34_ or the invariant chain (1 × 10^5^/well eAPCs, 90 Gy) were used to stimulate the TCL in 96-well round bottom plates for 4 days.

In addition, a Bet v 1-specific TCL was generated from PBMCs of an HLA-DRB1*01 and HLA-DRB1*03-specific Bet v 1 allergic donor and labeled with CFSE. Engineered APCs bearing the respective MHCII were exogenously loaded with 0.5 μg/mL Bet v 1_142–153_ in the presence of 100 μM AdEtOH for 5 h. After washing and irradiation (60 Gy) 3 × 10^5^/well eAPCs were used to stimulate the TCL in 96-well flat bottom plates for 5 days. Following stimulation, cells were harvested and stained with a PE-labeled CD4-mAb. Proliferation of TCL was assessed by flow cytometry and all experiments were performed in duplicate.

### qPCR

mRNA expression of HLA-DR, HLA-DM, HLA-DO and Cathepsin S using the Art v 1 eAPCs was measured by qPCR using primers described previously^26^.

### Statistics

Fold induction was calculated as the ratio of gMFI of stimulator cells plus antigen and gMFI of stimulator cells alone from duplicate or triplicate wells. In general, medians of fold induction are shown. Statistical analysis was performed with GraphPad Prism (version 5, GraphPad Software, Inc., La Jolla, CA). Two-sided paired t-tests (Mann-Whitney test), one-way ANOVA followed by Tukey’s posthoc tests and two-way ANOVA were conducted (ns … not significant, *P ≤ 0.05, **P ≤ 0.01, ***P ≤ 0.001).

## Additional Information

**How to cite this article**: Rosskopf, S. *et al.* Creation of an engineered APC system to explore and optimize the presentation of immunodominant peptides of major allergens. *Sci. Rep.* **6**, 31580; doi: 10.1038/srep31580 (2016).

## Supplementary Material

Supplementary Information
