# Supplementary material for: Creation of an engineered APC system to explore and optimize the presentation of immunodominant peptides of major allergens
Source: Sci Rep. 2016 Aug 19;6:31580. doi: 10.1038/srep31580 (PMC4990899; doi:10.1038/srep31580)
Supplement: Supplementary Information [file srep31580-s1.doc]

**Creation of an engineered APC system to explore and optimize the presentation of immunodominant peptides of major allergens**

Sandra Rosskopf^1^, Sabrina Jutz^1^, Alina Neunkirchner^1^, Martín R. Candia^1^, Beatrice Jahn-Schmid^2^, Barbara Bohle^2^, Winfried F. Pickl^1^, Peter Steinberger^1*^

^1^Institute of Immunology, Center for Pathophysiology, Infectiology and Immunology, Medical University of Vienna, Vienna, Austria

^2^Department of Pathophysiology and Allergy Research, Center for Pathophysiology, Infectiology and Immunology, Medical University of Vienna, Vienna, Austria

^*^Corresponding author, peter.steinberger@meduniwien.ac.at

**Supplementary Figure 1 - Expression analysis of eAPCs and effect of coinhibition:**

1. Expression analysis of MHCII, CD80 and invariant chain on eAPC. Open histograms: K562 cell line; filled histograms: indicated stimulator cells.
2. Expression analysis of invariant chain (CD74) on eAPC. Open histograms: isotype control; filled histograms: CD74.
3. Expression of PD-1 on T cell transduced Jurkat reporter cells and PD-L1 and PD-L2 on eAPC. PD-1 was expressed on Art v 1 and Bet v 1 reporter cells and PD-L1 and PD-L2 was expressed on eAPC. The reporter cells, eAPCs and respective control cells were stained for expression of PD-1, PD-L1 and PD-L2 as indicated.
4. Effect of PD-1 coinhibition on Art v 1 and Bet v 1 reporter activation. PD-1 expressing reporter cells were stimulated with eAPCs expressing fusion proteins of allergenic peptides with invariant chain constructs. Using PD-L1 or PD-L2 expressing eAPC, the effect of PD-1 ligation on allergen-specific reporter activation was assessed. A blocking antibody to PD-1 (Ab) was used to revert PD-1 mediated inhibition. Means of fold induction are shown for duplicate values and medians of fold induction of four experiments is indicated as line. Statistics by one-way ANOVA, followed by Tukey’s multiple comparison post-test (* P≤0.05; ** P≤0.01; ***P≤0.001; ns not significant).

**Supplementary Figure 2 - Expression analysis of eAPCs and assessment of allergenic peptide preloading:**

1. Expression analysis of MHCII, CD80 and invariant chain on eAPC expressing indicated fusion proteins of allergenic peptides with the invariant chain. Open histograms: K562 cell line; filled histograms: indicated stimulator cells.
2. Expression analysis of MHCII, CD80 and invariant chain of the K562 stimulator cells used for the competition assays. Open histograms: K562 cell line; filled histograms: indicated stimulator cells.
3. Evaluation of loading strategies with exogenous peptide. Allergen-specific T cell reporter cells were stimulated with eAPC in presence of allergenic peptides or with eAPC preloaded with peptide for 3 hours followed by a washing step. The effects of the non-peptide MLEs AdEtOH and p-CP were analyzed under both conditions. The mean of fold induction is shown for duplicate values and the median of fold induction of multiple experiments is indicated as line. Statistics by two-way ANOVA, followed by Bonferroni post-test (*P ≤ 0.05; **P ≤ 0.01; ***P ≤ 0.001; ns not significant).

**Supplementary Figure 3 - Expression analysis of used APCs and evaluation of surface HLA-DM:**

1. Expression analysis of MHCII, CD80 and CD58 of the used DR7 EBV cell lines. EBV1 and EBV2 are homozygous or heterozygous for HLA-DR7, respectively. Open histograms: isotype control; filled histograms: indicated eAPCs.
2. Expression analysis of MHCII, CD80, HLA-DM and Bet v 1 on eAPC. Open histograms: K562 cell line; filled histograms: indicated stimulator cells.
3. Evaluation of surface HLA-DM on allergenic peptide presentation. Bet v 1 reporter cells were stimulated with eAPC preloaded with 0.1 μg/mL Bet v 1_142-153_ peptide for 5 hours in presence or absence of an HLA-DM antibody (5 μg/mL) and sodium azide (4 mM NaN_3_, endocytosis inhibitor) followed by a washing step (left panel). In addition, eAPC preloaded with 0.1 μg/mL or 1 μg/mL Bet v 1_142-153_ peptide for 5 hours in presence or absence of chloroquine CQ were used for cocultures (right panel). The mean of fold induction is shown for duplicate values. Experiments were repeated with similar outcome.

**Supplementary Table 1:** Analysis of mRNA expression in eAPC of molecules involved in the MHCII antigen processing pathway. Cq values (quantification cycle) of qPCR experiments are shown. Lower Cq values correlate with higher target mRNA expression.
